# Outbreak of multidrug-resistant *Klebsiella pneumoniae* carrying *qnrB1* and *bla*_*CTX-M15*_ in a French intensive care unit

**DOI:** 10.1186/2110-5820-3-18

**Published:** 2013-07-01

**Authors:** Nathalie Filippa, Anne Carricajo, Florence Grattard, Pascal Fascia, Faten El Sayed, Jean Pierre Defilippis, Philippe Berthelot, Gerald Aubert

**Affiliations:** 1 Laboratory of Antibiology, University Hospital of Saint-Etienne, 42055 Saint-Etienne, France; 2 Bacteriology and Virology Laboratories, University Hospital of Saint-Etienne, Saint-Etienne, France; 3 Infection control unit, University Hospital of Saint-Etienne, Saint-Etienne, France; 4 Intensive care unit, Clinique Mutualiste of Saint-Etienne, Saint-Etienne, France

**Keywords:** Outbreak, *Klebsiella pneumoniae*, Extended-spectrum beta-lactamase, Intensive care unit, Screening, Quinolone resistance

## Abstract

**Background:**

The prevalence of extended-spectrum beta-lactamase (ESBL)-producing *Enterobacteriaceae* is increasing globally and is a major clinical concern. Between June 2008 and September 2009, 4% of patients in an intensive care unit (ICU) were found to be colonized or infected by strains of *Klebsiella pneumoniae* multiresistant to ceftazidime, ciprofloxacin, and tobramycin; an investigation was initiated and isolates were characterized by molecular typing and resistance patterns.

**Methods:**

Antibiotic susceptibilities were determined by Vitek2^®^, Etest^®^, and agar dilution. Gene encoding beta-lactamases and plasmid-mediated quinolone resistance PMQR determinants (*qnr, aac(6′)-Ib*) were characterized by PCR, sequencing, and transfer assays. DiversiLab^®^ fingerprints were used to study the relatedness of isolates.

**Results:**

Fourteen isolates co-expressing *bla*_*CTX*-M15_, *qnrB1*, and *aac(6′)-Ib-cr* were identified. Genotypic analysis of these isolates identified 12 clonally related strains recovered from 10 patients. The increased prevalence of *bla*_*CTX*-M15_-*qnrB1*-*aac(6′)-Ib-cr*-producing *K. pneumoniae* coincided with the presence in the ICU of a patient originally from Nigeria. This patient was infected by a strain not clonally related to the others but harbouring *qnrB1* and *aac(6′)-Ib-cr* genes, a finding not hitherto observed in France. We suspected transmission of resistance plasmids followed by rapid dissemination of the multiresistant *K. pneumoniae* clone by cross-transmission.

**Conclusion:**

This study highlights the importance of microbiological screening for multidrug-resistant strains in ICUs, particularly among patients from regions in which multidrug-resistant bacteria are known to exist.

## Background

The increasing prevalence of infections caused by multidrug-resistant organisms is a cause for grave concern among healthcare professionals, in particular within intensive care units (ICU). The wide spread of extended-spectrum beta-lactamase (ESBL)-producing *Enterobacteriaceae* is increasing globally and poses a major clinical problem in many countries [1]. Plasmid-mediated quinolone resistance (PMQR), especially involving Qnr proteins and the aminoglycoside acetyltransferase variant determinant (AAC(6′)-Ib-cr), has emerged and is now described worldwide [2]. PMQR determinants are generally reported in association with ESBL-producing enterobacteria, in particular the CTX-M type [2,3]. Herein, we describe the investigation of an outbreak in a French ICU of a strain of *Klebsiella pneumoniae* producing QRN-B and CTX-M-15-type enzymes.

## Methods

Between June 2008 and September 2009, the incidence of colonization or infection with *K. pneumoniae* strains multiresistant to ceftazidime (MIC > 4 mg/l), ciprofloxacin (MIC > 1 mg/L), and tobramycin (MIC > 4 mg/l) increased to 4% in the medical ICU of the Clinique Mutualiste, a 195-bed hospital centre equipped with 12 ICU beds (220 patients admitted annually). The medical files and microbiological data of patients colonized or infected with *K. pneumoniae* were reviewed. PCR amplification of *bla*_CTX-M_, *aac(6′)-Ib, qnrA*, *qnrB*, and *qnrS* sequences was performed as described by other authors [2,4,5]. All PCR products positive for *aac(6′)-Ib* were further analyzed by digestion with *Bts*CI (New England Biolabs, Ipswich, MA) to identify *aac(6′)-Ib-cr*, which lacks the *Bts*CI restriction site found in the wild-type gene [3]. All other PCR products obtained were sequenced. Transferability of *qnr* and *bla-*_CTX-M_ genes was studied by means of a conjugation assay using streptomycin-resistant *E. coli* J53 as the recipient. After 40 minutes of incubation, mating mixtures were plated onto agar containing streptomycin (100 mg/l) and ceftazidime (1 mg/l). The presence of *qnr* and *bla*_*CTX-M*_ in the transconjugants was confirmed by PCR as described above. Clonal relationships also were investigated using the DiversiLab^®^ fingerprinting system (bioMérieux), a commercially available repetitive-element (rep)-PCR tool successfully used for the typing of ESBL-producing *E. coli* isolates [6]. This study was conducted only on bacterial samples without changing the medical care of patients. It does not therefore fall within the French law on biomedical research and has not received notice of an ethics committee.

## Results and Discussion

**Figure 1:**
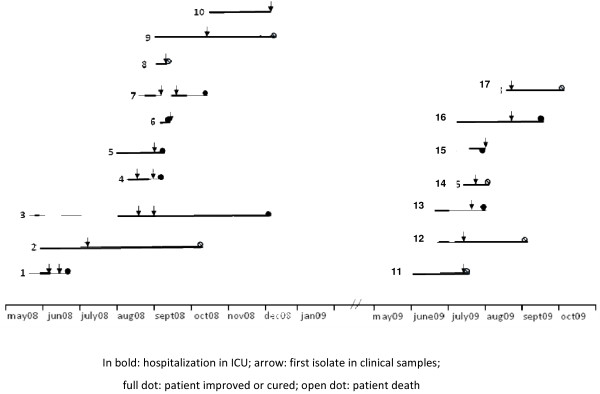
Length of stay in ICU for patients implicated in the outbreak hospitalization in ICU; arrow: first isolate in clinical samples; full dot: patient improved or cured; open dot: patient death.

**Figure 2:**
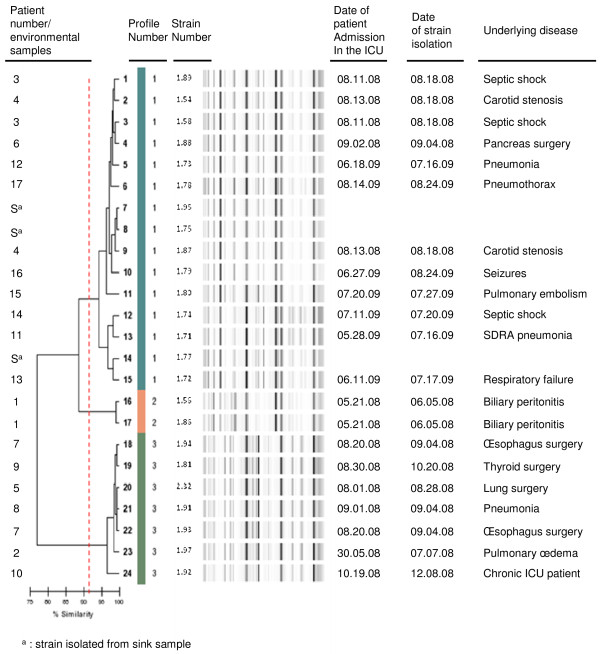
**Dendrogram analysis and virtual gel images of DiversiLab rep-PCR fingerprinting system (bioMérieux) of the 24 multiresistant *Klebsiella pneumoniae *strains isolated from 17 patients and 3 sinks (strain Nos 1.95, 1.75, and 1.77).**

The outbreak exhibited two major peaks: one in the summer of 2008 and another in the summer of 2009; no clinical cases were noted before June 2008, between these two periods, or after September 2009 (Figure 1) (Additional file 1: Figure S1). During the 15-month period of the outbreak, 17 patients (aged 37-86 years) exhibited a total of 21 isolates (Figure 2). Following identification of the outbreak, control measures were reinforced. Patients were placed in a specific area of the ward under the supervision of specially assigned staff members in order to reduce the risk of cross-transmission. During the second summer outbreak in 2009, all ICU sinks were screened for the presence of multidrug-resistant bacteria. Three sinks were found to be positive for CAZ-CIP-TM resistant *K. pneumoniae*: two in patient’s rooms and one in the nurses’ room. Fourteen of the 21 clinical CAZ-CIP-TM-resistant *K. pneumoniae* isolates were positive for bla_CTX*-M15*,_*qnrB1*, and *aac(6′)-Ib-cr* genes. Of the 14 *K. pneumoniae* strains harbouring *aac(6′)-Ib-cr* and *qnrB1* genes isolated from 11 patients, 12 displayed the same DiversiLab^®^ pattern (pattern 1, Figure 2), whereas 2 isolates from patient 1 showed a distinct profile (pattern 2, Figure 2). Furthermore, molecular typing confirmed that the three isolates collected from the sinks belonged to the same cluster (pattern 1, Figure 2). The first isolates (patient 1, strains 1.56 and 1.86) of *K. pneumoniae* harbouring *qnrB1* and *aac(6′)-Ib-cr* gene were recovered from a patient from Nigeria who was admitted for biliary peritonitis. This patient, infected with a strain of *K. pneumoniae* expressing a QnrB1 protein, may be considered the index case. We suspected plasmid transfer from a Nigerian *K. pneumoniae* strain to another *K. pneumoniae* strain, and conferring high-level resistance to antimicrobial drugs. The arguments in favour of this hypothesis were as follows: (i) no cases of infection by multidrug-resistant *K. pneumoniae* had been reported before admission of the suspected index patient, (ii) *QnrB1* had been described previously in Africa but not in France [7-9], (iii) *E. coli* transconjugants with *qnr* and *bla-*_CTX-M_ gene were obtained from this African *K. pneumoniae* strain, and (iv) the outbreak was terminated after eradication of potential reservoirs in the environment coupled with increased hygiene measures. *Klebsiella* infections may have spread rapidly as a result of environmental contamination (medical devices, soap, and disinfectants) or cross-transmission via the hands of healthcare workers [10]. In our study, epidemic *K. pneumoniae* strains were isolated from both patients and sinks (patients’ rooms and the nurses’ room), suggesting that contaminated water points could act as a secondary reservoir with a risk of contamination during handwashing. After reinforcement of hygiene measures, including cleaning of the environment within unit and replacement of the sinks, the outbreak ended. In the ICU, prior to this outbreak, no active surveillance of multiresistant bacteria was performed. However, such surveillance with isolation should form an active component of infection-control bundles to prevent the proliferation of multiresistant bacteria [11].

## Conclusion

In conclusion, we report an outbreak in a French ICU of a multiresistant *K. pneumoniae* strain possibly introduced from Africa. In France, very recent recommendations call for routine screening for multidrug-resistant bacteria in patients admitted to ICUs, particularly those repatriated from abroad (http://www.sf2h.net) (http://hcsp.fr). In practice, rectal swabbing or stool culture must be performed for such patients, with concomitant implementation of additional contact precautions to prevent cross-transmission. The results of our investigation clearly support the value of these new recommendations in preventing the diffusion of multiresistant strains among debilitated patients.

## Competing interests

The authors declare that they have no competing interests.

## Authors’ contributions

The authors carried out the molecular genetic studies, participated in the sequence alignment and drafted the manuscript. All authors read and approved the final manuscript.

## Supplementary Material

Additional file 1: Figure S1 AP PCR Result. Click here for file
