# Supplementary material for: Outbreak of multidrug-resistant Klebsiella pneumoniae carrying qnrB1 and blaCTX-M15 in a French intensive care unit
Source: Ann Intensive Care. 2013 Jul 1;3:18. doi: 10.1186/2110-5820-3-18 (PMC3707830; doi:10.1186/2110-5820-3-18)
Supplement: Additional file 1: Figure S1 — AP PCR Result. [file 2110-5820-3-18-S1.doc]

**AP PCR Result (additional data)**

**Material and method**

Clonal relationships were also investigated using arbitrarily primed polymerase chain reaction (AP-PCR), according to a standardized method (1). The profiles of strains isolated during the course of the outbreak were compared with unrelated epidemiologically strains using 2 different primers (no. 1: 5’-AACGCGCAA-3’ and no. 2: 5’-GGTGGTGGCT-3’) (2, 3). Strains exhibiting more than one band difference with each of the primers were defined as non related as described by Wang *et al*. (1).

**References**

1. Wang G, Whittam TS, Berg CM, Berg DE. RAPD (arbitrary primer) PCR is more sensitive than multilocus enzyme electrophoresis for distinguishing related bacterial strains. *Nucleic Acids Res* 1993; **21**: 5930-3.
2. Berthelot P, Grattard F, Patural H, Ros A, Jelassi-Saoudin H, Pozzetto B, Teyssier G, Lucht F. Nosocomial colonization of premature babies with *Klebsiella oxytoca*: probable role of enteral feeding procedure in transmission and control of the outbreak with the use of gloves. *Infect Control Hosp Epidemiol* 2001; **22**: 148-51.
3. Grattard F, Pozzetto B, Tabard L, Petit M, Ros A, Gaudin O. Characterization of nosocomial strains of enterobacter aerogenes by arbitrarily primed-PCR analysis and ribotyping. *Infect Control Hosp Epidemiol* 1995; **16**: 224-30.

**Results :** As shown in figure 1, molecular fingerprinting were identical with 2 primers in AP PCR for the QNRB and CTX-M producing strains except for the patient supposed to be the index case. Thus this confirmed the results obtained with diversilab® technique

**strain n° M 1.56 1.89 1.87 NR1 NR2 NR3 NR4 NR5 1.93 1.94 1.92 1.72 1.74 1.95 T- M**


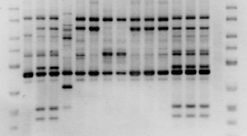


**A B B D C C E E C C C B B B**

**M 1.56 1.89 1.87 NR1 NR2 NR3 NR4 NR5 1.93 1.94 1 .92 1.72 1.74 1.95 T- M**


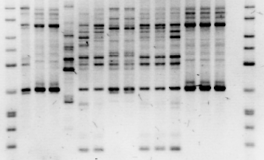


**AP-PCR pattern A B B D C C E E C C C B B B**

***aac(6’)-Ib* cr cr** cr Wt  **cr wt cr cr cr cr cr cr cr cr**

***qnr* B1 B1 B1 - - - - - - - - B1 B1 B1**

***blaCTX-M* 15 15 15 - - 14 - - - - - 15 15 15**

**M**= molecular weight markers; **wt** = wild type *aac(6’)-Ib*; **cr** = *aac(6’)-Ib-cr*

Figure 1 : AP-PCR typing with primer 1 (top) and primer 2 (low) of *Klebsiella Pneumoniae* strains of infected or colonized patients - strains 1.56: isolated in the index case patients; strains 1.89, 1.87, 1.72, 1.74: isolated from the patients implicated in the outbreak; strain 1.95 isolated from a sink; strains 1.93, 1.94, 1.92: isolated from patients not implicated in the outbreak; strains NR1, NR2, NR3, NR4 and NR5: no related *K. pneumonia* strains isolated in other ICU of our hospital.
